# Impact of information and communication software on multiprofessional team collaboration in outpatient palliative care – a qualitative study on providers’ perspectives

**DOI:** 10.1186/s12904-023-01141-4

**Published:** 2023-03-08

**Authors:** Anastasia Suslow, Chantal Giehl, Jannis Hergesell, Horst Christian Vollmar, Ina Otte

**Affiliations:** 1 Institute of General Practice and Family Medicine (AM RUB), Ruhr University Bochum, Universitätsstraße 150, 44801 Bochum, Germany; 2 Research Network of Old-Age Provision, Deutsche Rentenversicherung Bund, Ruhrstraße 2, 10709 Berlin, Germany

**Keywords:** Palliative care, Palliative medicine, Multiprofessional teams, Communication, Information and communication technology, Qualitative research, Team collaboration

## Abstract

**Background:**

The communication processes between different stakeholders in outpatient palliative care face challenges when multiprofessional teams want to keep each other updated on patient information. Meanwhile, the software market offers different tools to connect these teams in real-time to improve communication. In the research project ADAPTIVE (Impact of Digital Technologies in Palliative Care), we investigated how information and communication technology affects collaboration and work in multiprofessional teams and what advantages and disadvantages the use of said software might entail.

**Methods:**

We conducted 26 semi-structured interviews between August and November 2020 with general practitioners (n = 8), palliative care nurses (n = 17), and a pharmacist (n = 1). They were conducted in a hybrid format, meaning that both face-to-face interviews and telephone interviews were carried out. Subsequently, we analyzed the interviews following the qualitative content analysis according to Kuckartz.

**Results:**

Information and communication software has the potential to enable faster communication and delegation of tasks and to simplify communication and task management between providers. Furthermore, it creates the opportunity to decrease unnecessary supervision of duties and responsibilities for physicians in multiprofessional teams. Therefore, it allows facilitating the collaboration between multiprofessional teams that work independently of each other but care for the same patients. All providers have the same knowledge about their patients without time-consuming coordination such as phone calls or search processes in paper documentation. On the other hand, mishandling, poor Internet connection, and unfamiliarity with various features can diminish these benefits.

**Conclusion:**

Even though the use of such software offers many advantages, these advantages only reveal themselves if the software is used as it was intended by the developers. Misuse and unawareness of the individual functions can lead to the full potential not being realized. The software developers frequently offer specialized training, and the multiprofessional teams should utilize that to improve team communication, facilitate tasks, and allow physicians to delegate tasks.

**Trial registration:**

The study is registered in the German Clinical Trials Register (DRKS): https://www.drks.de/drks_web/navigate.do?navigationId=trial.HTML&TRIAL_ID=DRKS00021603 (Registration number: DRKS00021603; date of first registration: 02/07/2020).

## Background

The main goal of palliative care contains relief from pain, support for the patients and their families, concerning offering assistance for psychological, social, and spiritual suffering [1]. To do so, it is necessary that various stakeholders work together in a multiprofessional team, which is characterized as a collaboration between different occupational groups that take care of the same patients independently [2]. In German palliative care settings, those different groups are general practitioners, nurses, advanced practice nurses, and care managers who coordinate palliative care by phone and face-to-face by interacting with providers and patients, exchanging patient data, and providing social support for patients and their relatives [3]. Palliative care in Germany is distinguished into general and specialized palliative care. In addition to inpatient care, both are provided on an outpatient basis by nursing and hospice services, nursing homes, general hospital wards, as well as the stakeholders, mentioned above. However, an important component, is that general practitioners work closely with palliative care physicians, contribute their knowledge, and, at best, have a long-lasting relationship with the patients. The patients have the advantage of being cared for in their last phase of life by someone they know and trust. If this care is not sufficient, specially trained providers are brought in to make the last phase of life as comfortable as possible, preferably at home [4].

Furthermore, a broad variety of different stakeholders complements basic team and additional stakeholders, like physical therapists, social workers, psychologists, pastoral counselors, occupational therapists, art therapists, music therapists, nutritionists, and case managers [5–8]. In those teams, the different stakeholders are aligned with the needs of the patients and their relatives and provide the needed care [6]. As a consequence, the various stakeholders are required to work closely together to clarify and review care objectives and to define roles in which communication takes place in a structured and transparent manner [1, 8].

However, clarification is needed on who documents what kind of information and how it is communicated to other stakeholders [5]. The larger the team, the more challenging communication tends to be, and the connection between the various providers varies strongly [1, 9].

To overcome barriers such as potential information gaps due to the lack of frequent interaction with patients and other stakeholders [3], the use of information and communication technologies can provide a reasonable addition to simplify the communication in multiprofessional teams by bridging geographical distances and rapid exchange between the providers [9]. In addition, these technologies are expected to be supportive by allowing nurses to intervene sooner in the event of complications, even in the absence of a physician [8].

To address the above assumptions the ADAPTIVE project – Impact of Digital Technologies in Palliative Care – started in March 2020 to examine the impact of such technologies. Available research at that time was limited: while addressing how technologies can be beneficial to the elderly, these research teams were predominantly studying the geriatric field [10, 11]. So far the work with software that allows multiprofessional teams to communicate in real-time has not been investigated.

In this paper, we will focus on the following research questions: Which work-related changes entail a new communication method, and how does it improve teamwork? Additionally, if such software can affect team collaboration, can physicians delegate work more effectively and quickly?

## Methods

In order to be able to answer our research questions, we decided to conduct qualitative interviews with users of information and communication software. For this purpose, we first developed an interview guideline based on the literature, which we discussed within the team. This was followed by a pilot test run of the interview guideline with a colleague from the department. After that we incorporated the resulting feedback and were thus able to complete the guideline development. The semi-structured guideline addressed software use for exchanging data in general and specialized outpatient palliative care between different stakeholders For this purpose, the project team focused on one software called ISPC (Information System Palliative Care)^1^ in order to achieve comparable results. Contrary to other software, ISPC allows multiple providers operating independently to share information about their patients, even if they are not affiliated with the same hospital, for example.^2^ Other functions include documenting care, assigning tasks, and gathering all necessary information about patients, their condition, family members, and more.

The core topics of this interview guideline were (a) experiences with the software in everyday professional life (implementation); (b) daily use of the software and the associated data exchange, as well as the distribution of tasks in the team that arises as a result; (c) the question of personal perceptions of data protection; and (d) digitalization and telemedicine in general and in relation to Covid-19.

### Recruitment

Originally, recruitment was aimed at palliative care providers (outpatient palliative care nurses and physicians) in a local clinic, which recently implemented the software Information System Palliative Care (ISPC). To accomplish this, we were assisted by a contact person within the clinic who was available to support us throughout the recruitment process. However, due to Covid-19, the clinic reported overtime, sick notes, and a lack of time capacities to incorporate the software and to attend our research. Therefore, we could only conduct two interviews in this particular clinic. To continue our project, we broadened our recruitment strategy, contacted a palliative care network, and could gain additional nine interviews with different outpatient stakeholders working together in a regional and multidisciplinary team. This palliative care network also worked closely together with the previously selected clinic and was brought to our attention by the clinic’s contact person. As a result, we were able to create a broad contact network and recruit additional participants. Since we did not reach data saturation, we then further contacted the software developer, who transmitted our concern to all 4.000 users. Interested providers were then able to contact us allowing us to conduct fifteen additional interviews. Overall, the providers we contacted often reported that due to increased sick leave (not specified if this was related to Covid-19), additional worktime, and lack of capacity, there was no time or no interest in a one-hour interview.

For this reason, we had to postpone the recruitment period by two months. In the end, we could target the time frame even though we had to contact more providers than initially planned. Summarized, we combined theoretical with empirical sampling [12] in a purposeful sampling strategy by targeting our concerns to palliative care providers.

### Interview conduction

The first interview, using a semi-structured guideline [13], took place in the summer of 2020 and should provide initial insight into the usage and trying out the new software. We examined how the software changes the nurses’ daily work routine and how it changes the interaction with the patients since the first interviews. Subsequently, a second interview with the same participants was planned and should have shown how the application developed after a routine settled down.

Due to Covid-19, the related lockdowns, and increased workload in nursing professions, we had to modify our approach and cancelled the second interview. In addition, we have already included all the questions planned for the second interview in the first guideline. The extended recruitment also allowed us to talk to participants who have been using the software for several years.

Five participants invited us to conduct the interviews in their work environment, the other 21 were conducted by phone. Every participant received an information sheet and an informed consent form, which they were asked to sign and return to us before the interview.

Finally, we interviewed 26 participants (physicians n = 8; palliative care nurses n = 17; pharmacist n = 1) with a duration of 45 to 60 min until we could observe data saturation [14].^3^ It is important to note that the interviewed palliative care nurses often acquired additional qualifications and performed the roles of other stakeholders, for example, by also serving as bereavement counsellors or wound managers. Among the physicians, there were also qualified palliative care physicians, general practitioners and even a psychologist.

### Analysis

The 26 interviews were conducted between August and November 2020, audio-recorded and transcribed verbatim. The interviews were then pseudonymized during the transcription process. Our study followed the qualitative content analysis according to Kuckartz [15] using the software MAXQDA [16]. Initially, we coded the data deductively based on the interview guideline. During the analysis process, we detected new inductive codes and evaluated them thematically. We reviewed and discussed the results among our core research team consisting of three persons (AS, CG, IO) with the following backgrounds: sociology, health services research, and medical ethics. One of them (AS) conducted the interviews and analyzed them in collaboration with the others. In case of discrepancies, these were critically discussed in a larger group with additional expertise (medicine, nursing science, public health) and revised if necessary.

## Results

The interviews provided a variety of interesting results which, due to their complexity, cannot all be discussed in one publication. For this reason, we limit ourselves in this manuscript to collaboration in a multiprofessional team.

Based on the analysis, we were able to capture the following three core findings, which include a focus on the different effects a new communication tool like ISPC has on the collaboration in multiprofessional teams in palliative care: delegation of tasks, improvement of team collaboration, and improvement of working conditions.

Furthermore, we can add that the software is primarily used in outpatient palliative care, as this software facilitates exchange during home visits by different teams. In the hospital where it has been used, the software was used as an “add-on” alongside the traditional documentation software to document palliative cases.

### Delegation of tasks

Physicians reported a broader variety of delegating tasks. The software simplifies the delegation of tasks by utilizing its accessibility for every provider involved. In addition, the software enables simple and quickly available task distribution with the help of so-called to-dos, including traceability of completion and written, traceable arrangement. The traceability of the documentation is granted, so that it can be recognized which person has distributed or ordered tasks, which person has completed them, as well as which data has been entered by whom. This provides judicial security (e.g., regarding the administration of medication):

4 *PCTP-03 – Palliative care nurse: “Well, what’s good is that you have the delegation […], i.e. the doctor’s order, right in the system. So what you were familiar with in the past […] we no longer have that, of course, because they document it immediately and it is also verifiable in the system in a data-proof manner, right? That is of course a great advantage.”*

*PCTP-02 – Palliative care nurse: “And above all, I can also directly carry out doctors’ orders that I can see online. If I tell myself I’ll only do it with an order, which I’m actually only allowed to do as a nurse. And of course, it’s much faster.”)*

*PA-06 – General practitioner: “Communication is much easier. In other words, I don’t have to make phone calls about every little thing. I can delegate things and see that they are done. So I don’t have to laboriously follow up again, ask, ‘Did you do that too?”*

Altogether, we can see that information and communication technologies simplify the communication and task management between the providers and decrease unnecessary supervision of duties and responsibilities.

### Improvement of team collaboration

As we could see, participants essentially used the main function of the software, and it was well accepted that easy transfer of patient data to other providers is possible if they also use the same software. Patient data can be easily shared instead of having to be obtained in a time-consuming manner:

*PA-03 – General practitioner: “We can exchange ideas, right? The best example: my colleague went on vacation and handed over his patients to me. So the, what should I say, problem children. And then, when there were actually corresponding inquiries, I was able to see his entries immediately, to understand them, and to see them more or less immediately without any, what should I say? Loss in communication or in empathy immediately to continue his work with these patients.”*

As well it is possible that all providers share the same knowledge:

*PCTA-02 – Palliative care doctor: “[…] We have the possibility via the software to see at all times: who was involved and when, and what has changed in the perception? And even if, under certain circumstances, we are no longer responsible for the support, for example, if we transfer the patient to an inpatient setting or if the patient has died, we still have the opportunity to check this evaluation via the individual case documentation: How did it develop? What was our perception at the beginning? How is it now? So I think this is an important tool for the quality of the work.”*

Additionally, the pharmacist we interviewed confirmed the benefits a web-based rapid exchange provides:

*AP-01 – Pharmacist: “And, of course, there are also some intolerances to medications, which are also entered. That’s another double check for me, to see: Has this now perhaps been prescribed by mistake? […] We sometimes have cases where patients do not want to accept this box at all […]. And then they say ‘I don’t want to’, then I can make a note ‘Patient has refused to accept’. If the doctor has to go out at night and the things [medicaments] are not there, that would be annoying […].”*

### Improvement of working conditions

The simultaneous access of different providers allows simple communication between them and avoids additional often time-consuming work (e.g., secondary phone calls). Paper documentation is no longer necessary – the software eliminates the need to search through paper folders and facilitates the retrieval of information:

*HP-05 – Hospice nurse: “ISPC has made things much easier, so the general level of information in the team is higher as a result. And you can also trust that your colleagues know what’s going on. And you can also just rely on it and say, ‘Well, just take a look at ISPC,‘ right? Especially because we are also on call and then one colleague is responsible for all our patients at night.”*

This yields improved collaboration among stakeholders:

*PA-03 – General practitioner: “Yes, the collaboration between us has improved significantly in so far as we can enter our data there in the traditional way and, if I’m on the road somewhere or whatever, I can access it immediately with my terminal device and see if I’m on call, let’s say and I’m called to a patient I don’t know personally, then I can see very quickly what’s happened there without having to call my colleague first (…). And the improvements keep coming up - whether it’s the standard of medications or the faster contact with the pharmacy - that’s going wonderfully, has improved significantly the communication with each other.”*

Since the software is web-based, it can be used regardless of location, and users can access it from anywhere – whether an internet connection is available, and the software is used on a laptop, smartphone, or tablet. However, if there is no internet, poor access, or the software is only used on a desktop computer, slow or no mobile access to the data is possible:

*PCTP-03 – Palliative care nurse: “And that is 1:1 documentation, I can now read what my colleague agreed with the patient 10 minutes ago 50 kilometers away. That’s great.”*

*PCTP-04 – Palliative care nurse: “And being able to network was of course a big achievement. And the major advance was that it was web-enabled, that I could access it from anywhere, that the data was secure, and that I was always up to date, even if I wasn’t going to meet in person. And that, I guess, was convincing right from the start.”*

Based on the information in the software, communication with patients and relatives can be facilitated; since atmospheric and other relevant information can be entered in addition to medical parameters (e.g., access routes; aggressive pets; difficult family situations, etc.).

*HP-01 – Hospice nurse: “And that is incredibly valuable, so there has really been a change compared to before because we often had very little information about the patient. Or it is always like this: What they tell us is perhaps something different than what they tell the doctor, and sometimes it is simply important to know more about this medical situation.”*

It also streamlines vacation handoffs between physicians, making it easy to refer to what patients need:

*PA-03 – General practitioner: “We had this case with vacation replacement, a very seriously ill 19-year-old girl. Mr. [name] was the palliative physician primarily responsible but then went on vacation. I was in charge and was able to see extremely quickly on the basis of the program - or on the basis of the entries, I might say - where the problems were. Not only cancer and the resulting complaints, but also the social environment, which was difficult. Who is my contact person? Where could there be problems in communication, also in the understanding of the disease? No, these are definitely the advantages of the program.”*

The wealth of information in the software enables nurses to act quickly, even in acute situations, as all relevant information is ideally already available:

*PCTP-02 – Palliative care nurse: “And, of course, it’s much faster. So for our office staff, who of course have no medical training, if I see that I need it now and I need it right now, then I don’t have to wait three hours until I come back to the office to write a prescription, but I can do it all without a phone. Even if I can’t reach anyone right now, I can enter it into the system and by the time I get back, it’s already processed and ready.”*

In addition, the information is also immediately transcribed and can thus be read without any detours or communication problems:

*PCTP-06 – Palliative care nurse: “Well, there are advantages, of course, if I’m at the patient’s home at night, for example, and on the basis of our needs plan, which is on-site as a paper, the medication is simply no longer sufficient, and I call the palliative care physician and he makes a change, which he immediately enters in the ISPC, and I can then login and check within a few minutes and am then immediately ready to act. That’s great, of course. Of course, you can also do it by phone, but especially when it comes to narcotics, it’s nice to have it written down.”*

Finally: The nurses appreciate that instructions are written down and that work steps remain comprehensible:

*PCTP-03 – Palliative care nurse: “Well, what’s good is that you have the delegation, i.e., the medical order, right in the system. So, what you know from the past, when you agreed on things by phone with the medical colleagues, that you have to have it countersigned the next time they are there, or that you have to document the verbal order and read it out again and document, read out and approved. Of course, we don’t have that anymore, because they document it immediately and it is also verifiable in the system.”*

Altogether, we can see that the software enables faster communication and delegation of tasks. Therefore, it allows facilitating the collaboration between multiprofessional teams that work independently of each other but care for the same patients. All providers have the same knowledge about their patients without having to dial each other or search for the information in disorganized paper documentation.

On the other hand, there were also difficulties in using the software, so the interviewed participants sometimes did not know that they could also use the software on mobile devices, or the internet connection, especially in rural areas, was not always available.

*PfP-01 – Nurse: “It’s a pity that we only have access from the office. It would be better if the employees who are directly on-site with the patient could use ISPC immediately.”*

*PCPT-01 – Palliative care nurse: “Well, sometimes we don’t have a stable Internet connection here, and when it breaks down in the middle of documenting or with the iPad […]. And if I then have to document a second time because it didn’t take over the first time or didn’t save properly - yes, that’s annoying sometimes.”*

These unexpected negative aspects show the software’s flaws which point to operating errors. The poor internet connectivity can also be bypassed by using an offline mode. The reason that we did not expect these results is that the software developers offer training on how to use the software, and we assumed that all users would take advantage of this training.

In addition, the physicians from the clinic and a nurse from a private practice mentioned that another internal documentation software was also used, resulting in double documentation.

In conclusion, there were not many negative aspects, as participants described the software as self-explanatory and easy to use. When asked about data protection, no negative aspects were mentioned either, as the software was perceived as consistently secure.

Nonetheless, all participants reported regular meetings with each other to actively share ongoing cases, even when tasks and procedures could be communicated through the software.

## Discussion

As we have seen, multiprofessional cooperation in palliative care is central to appropriate patient care. We were thus able to indicate that communication software can contribute to a better delegation of tasks and improved collaboration. This can lead to enhanced care. To support this statement, the perspective of patients and family members could be included in further studies. This allows the topic to be viewed holistically.

We were able to identify several enabling factors, allowing multiprofessional teams to administer palliative care faster, safer, and potentially better by: (1) easier and safer delegation of tasks, (2) improved working conditions, and (3) a better collaboration within teams.

### Delegation of tasks

General practitioners often prefer to work in teams of palliative care stakeholders also because of a higher quality of care [17]. This involves defining the goals and tasks of the individual providers and, if necessary, delegating tasks [1]. Particularly in outpatient palliative care, when nurses are with the patient, and an emergency situation arises, it can relieve the burden on all those involved if, for example, medications are already recorded in advance using communication software [8]. In this case, the general practitioner or palliative care physician, who may be unavailable in a critical situation (e.g., at weekends or during the night), can delegate the administration of medication to nurses by consulting with them in advance and recording the information in the software. In these situations, it may be possible for the nurse to make decisions and administers medication. Nursing staff, who are only allowed to administer medication with a written order, feels legally and ethically protected in their actions through documentation, as work steps remain precisely traceable. But also, general practitioners, who are responsible for many patients due to understaffing can be relieved by administering tasks. However, this requires a “culture of conversation with transparent structured communication channels” [3, 8].

### Improvement of team collaboration

As reported in the section above, documentation in an information and communication software offers the advantage that said documentation is secured. This protection is also provided by the fact that instructions and information are digitally recorded, which minimizes the margin for error due to illegible handwriting or telephone calls with insufficient sound quality and the resulting miscommunication. And all providers are on the same level due to the fast exchange and have all information at hand.

The elimination of many phone calls can result in faster and easier transmission and execution of tasks. Problematic accessibility is also eliminated by real-time transmission, provided there is a stable internet connection. The resulting valuable time can be used for patient care and thus improve the quality of care.

Furthermore, this fast and continuous documentation offers the possibility to examine and reflect on work processes systematically and thus to consider together in the team what could be improved under certain circumstances, which can also affect the collaborative work and the quality of care.

### Improvement of working conditions

Nurses have more perceived independence of action and greater scope of action because of being able to administer medications in a legally secure manner once they have been entered into the software. This can relieve the pressure of having to immediately contact a physician who may not be easily available at night or on weekends. Overall, all providers can enter a situation more prepared if atmospheric information is also documented, as described in the quotes.

Consequently, communication at the same level can develop in the team and information does not have to be obtained laboriously if everyone uses the software to the same extent. As shown in the results section, underutilization due to lack of knowledge, disregarding training offers, and inadequate infrastructure (e.g., poor rural internet connection) can directly eliminate the benefits of such software. Therefore, non-documentation could again lead to additional work and, in the worst case, endanger patient care due to missing or incorrect information. If this type of communication is given and the software is used as it was conceived by the developers, the use of communication software facilitates communication between a physician and other providers. Everyone involved stays informed about their patients and contingency plans can already be filed, eliminating the need for difficult decision-making on the part of the nurses [18] resulting in an improvement in working conditions.

Compared to other documentation software, such as PalliDoc, the software we examined offers a web-based exchange of important information between all stakeholders. In multidisciplinary teams, purely internal documentation can result in information only arriving with a time delay and having to be obtained by faxes, but also by telephone calls.

The various programs are not compatible, so data cannot be transferred from one software to another. However, this means that practices and clinics may have to use several programs at the same time. On the one hand, the internal software that is used in the practice or clinic, but also a web-based software that is specified by the cooperation partners to be able to exchange information through it.

Since not all palliative care providers have yet switched entirely to ISPC for various reasons (e.g., continuing contracts with the original software), this results in a double burden.

## Strengths and limitations

The focus on one particular software (ISPC) may be considered a limitation of the study. While there is other software used in palliative care such as PalliDoc, these were not addressed in the interviews. However, this software used in outpatient palliative care is comparable to other products in that it offers its users the same features and options but with the extension to real-time communication and exchange between independent providers. The findings presented in this paper are inductively derived from the analysis of the participants’ experiences. It should be noted that these emerged from the interviews and therefore reflect the individual opinions of the participants. The physicians and nurses depicted here also take on the roles of other providers, using their additional qualifications to act as grief counselors or wound managers, for example. For this reason, the participants appear to be limited to physicians and nurses. Only one interview with a pharmacist was possible. The interview with the pharmacist showed how medication orders are processed through the software and subsequently delivered. The interviews with the physicians and nurses showed the same results. Furthermore, the team collaboration with pharmacists was not carried out in this regard by the participants.

In addition, the Covid-19 pandemic made recruitment difficult, and we had to deviate from our initially planned approach. The sampling was extended several times to recruit enough participants. The plan was to recruit within one clinic, which expanded to the region, the entire state, and ultimately throughout Germany. For this reason, we could not entirely compare the teamwork directly based on different team members to each other but had to rely on the participants’ statements. Furthermore, the participants had been using the software for different lengths of time. Some had been using it for eight years, while others had only been working with it for a few months. This also contradicts the original idea of investigating the development of software use within a team. On the other hand, this approach was also able to provide insights into long-term usage. But in the end, we could recognize in the data that similar statements were made in the interviews so that we could assume theoretical saturation.

However, this study provides important insights into the utilization patterns and consequences of providers’ use of communication software in outpatient palliative care. To date, there has been insufficient empirical data on this topic, so the results presented should be considered enriching.

## Conclusion

The qualitative interviews with general practitioners, palliative care nurses, and pharmacists were very saturated with data and showed that communication software illustrates an impact on multiprofessional collaboration. The main results from this qualitative study provide two main insights: (a) general practitioners can delegate more tasks and can thus be relieved in their work, and (b) stakeholder can share information more conveniently, frequently, and reliably, which results in improved multiprofessional team collaboration and improved working conditions in general.

Nonetheless, some participants stated that the software was only used internally as documentation software, without networking with other providers. Similarly, it was mentioned that not all providers are always up to date on entries. Furthermore, some participants mentioned that the software is not usable if there is no internet connection, even though the software offers an offline mode, and users can thus access the data even if there is no or a weak internet connection. Only if communication software is used properly, it can prevent information and supply disruptions and consequently improve team collaboration [8].

Most software publishers offer training to demonstrate and explain all the features of their products. This training should be used by the palliative care teams that are looking to improve their communication to take advantage of all the possibilities. In this way, mishandling can be minimized and palliative care teams can benefit from the advantages of this method of exchange.

## Data Availability

The datasets generated and/or analysed during the current study are not publicly available due to ensure the anonymity of the participants but are available from the corresponding author on reasonable request.

## Footnotes

1 For ease of reading, only the term ISPC will be used throughout this publication. Whenever communication software is mentioned in general, we retain the right to use the term software without referring to a specific application.

2 Even though there are other software providers, we chose this one to generate comparability on the one hand, but also to capture the communication possibility. The research team is not financially supported by the software developers and there is no conflict of interest.

3 More information regarding our experiences conducting qualitative research during a pandemic is available in an additional publication within this research project.

4 The original quotes were translated from German into English by the researchers.
